# The Clinical Significance of the Subtypes of Detrusor Overactivity: A Systematic Review

**DOI:** 10.1002/nau.70110

**Published:** 2025-07-03

**Authors:** Manisha Teji, Nicholas Raison, Nicholas Faure‐Walker

**Affiliations:** ^1^ GKT School of Medical Education, King's College London London UK; ^2^ School of Biomedical Engineering, King's College London London UK; ^3^ King's College Hospital NHS Foundation Trust London UK; ^4^ King's College London, Faculty of Life Sciences and Medicine London UK

**Keywords:** detrusor, DO, end fill, neurourology, overactivity, phasic, terminal

## Abstract

**Introduction:**

Detrusor overactivity (DO) is the “occurrence of detrusor contraction(s) during filling cystometry” [7] and can be “phasic” or “terminal” [26]. The aim of this systematic review was to establish whether there is any clinical significance between the different subtypes of DO in terms of demographics, underlying pathophysiology, symptoms, other urodynamic parameters and response to treatment.

**Methods:**

A systematic search was performed using PubMed, Cochrane, MEDLINE, Web of Science and EMBASE databases in February 2024, following the PRISMA guidelines. The Newcastle Ottawa critical appraisal tool was used to assess the risk of bias in the included studies.

**Results:**

The search identified 16 relevant studies including eight prospective and eight retrospective studies. Patients with terminal DO were found to be significantly older (*p *= 0.0003) and were more likely to have suffered spinal cord trauma (*p *= 0.04) compared to those with phasic DO. Patients with terminal DO were also found to have higher symptom scores and more likely to also suffer with incontinence than those with phasic DO. Patients with terminal DO were found to have smaller functional capacities than those with phasic DO on urodynamics. Success rates, following trans‐urethral resection of the prostate (TURP), were 69.2% for patients with phasic DO and 9.5% for those with terminal DO.

**Conclusion:**

Patients with terminal DO are more likely to be older, suffer with worse symptoms, have smaller bladder capacities and respond less favourably to treatment than those with phasic DO. Future studies incorporating DO should consider analysing these two sub‐types of DO as separate clinical entities.

## 1 Introduction

Detrusor overactivity (DO) is defined by the International Continence Society (ICS) as “the occurrence of detrusor contraction(s) during filling cystometry” [1]. It is a urodynamic finding. These contractions, which may be spontaneous or provoked, produce a wave form on the cystometrogram, of variable duration and amplitude. DO is found in approximately 10% of the population and in almost 80% of elderly patients undergoing urodynamic assessments [2]. DO can manifest clinically as urgency, frequency, and incontinence, leading to a significantly impaired quality of life for patients affected [3].

The ICS divides DO according to its underlying aetiology and to its form: In the 2018 ICS report on the terminology for the adult male lower urinary tract and pelvic floor symptoms and dysfunction, the aetiological sub‐types include “idiopathic” DO, “neurogenic” DO where there is an associated, relevant neurological disorder and non‐neurogenic (secondary) DO where there is a non‐neurological provoking factor such as bladder outlet obstruction or urinary tract infection [1]. The report states DO contractions can be “suppressed by the patient or uncontrollable” and that their form “may be phasic or terminal.” The 2018 ICS report on the terminology for adult neurogenic lower urinary tract dysfunction (NLTUD) defines “phasic” DO (PDO) as a “characteristic wave form” observed in detrusor overactivity (PDet) as the bladder fills; “terminal” DO (TDO), sometimes described as “end‐fill” DO, is defined as “involuntary detrusor contraction occurring near or at the maximum cystometric capacity, which cannot be suppressed, and results in incontinence or even reflex bladder emptying (reflex voiding)” [4].

In addition to phasic and terminal DO, the ICS has also recognised compound detrusor overactivity and sustained detrusor overactivity as distinct urodynamics patterns [1]. Compound DO is characterised by multiple detrusor contractions that occur with increasing amplitude during filling cystometry, while sustained DO refers to a prolonged involuntary detrusor contraction that does not return to baseline pressure. However, due to the limited studies specifically examining the clinical significance of these subtypes, the are not included in this review.

Definitions have evolved over the years and have now been standardised by the ICS [1, 5, 6, 7, 8]. Despite this, there is a paucity of literature establishing the clinical relevance of the different patterns of DO.

### 1.1 Definitions

This systematic review will incorporate defined terminology as described by the ICS to maintain consistency and precision across all discussions about detrusor overactivity [1]. Relevant definitions used in this review are summarised in Table 1.

**TABLE 1:** A table of defined terminology relevant to this systematic review [1].

Term	Definition
Detrusor overactivity	The occurrence of detrusor contraction(s) during filling cystometry. These contractions, which may be spontaneous or provoked, produce a wave form on the cystometrogram, of variable duration and amplitude. The contractions may be phasic or terminal (end fill). They may be suppressed by the patient or uncontrollable [1].
Phasic detrusor overactivity	A characteristic waveform that can occur during filling cystometry [1].
Terminal detrusor overactivity	A single involuntary detrusor contraction that cannot be suppressed and that occurs when the maximum bladder capacity is reached, resulting in urinary incontinence and often complete bladder emptying [1].
Neurogenic detrusor overactivity	Detrusor overactivity and evidence (history; visible or measurable deficit) of a relevant neurological disorder [1].
Idiopathic detrusor overactivity	Detrusor overactivity where there is no identifiable cause for involuntary detrusor contraction [1].
Compound detrusor overactivity	Phasic detrusor contraction with a subsequent increase in detrusor and base pressure with each subsequent contraction [1].
Sustained detrusor overactivity	Continuous detrusor contraction without returning to the detrusor resting pressure [1].
Overactive bladder	Urinary urgency, usually accompanied with increased daytime frequency and/or nocturnal, with urinary incontinence (OAB‐wet) or without (OAB‐dry), in the absence of urinary tract infection or other detectable disease.
Urgency	Complaint of sudden, compelling desire to pass urine which is difficult to defer.
Frequency	Complaint that voiding occurs more frequently than deemed normal by the individual (or caregivers).
Urinary incontinence	Complaint of involuntary loss of urine.
Urgency urinary incontinence	Complaint of involuntary loss of urine associated with urgency.
Urodynamics	Measurement of physiological parameters relevant to the functions of the lower urinary tract [1].

If a treatable, suspected underlying cause of DO, such as bladder outflow obstruction, is identified, patients often undergo treatment for obstruction in the first instance. Owing to the symptoms which DO normally leads to, patients with DO, who have received treatment for any rectifiable underlying cause, are normally managed with a variety of treatments ranging from anticholinergics and beta‐3 adrenoceptor agonists to intradetrusor injections of OnabotulinumtoxinA or neuromodulation [7, 8, 9]. While extensive research has investigated the effectiveness of these therapies on the general population with DO, there remains a notable gap in the literature currently, with few studies specifically assessing the clinical significance of PDO and TDO.

The aim of this systematic review is to ascertain any clinical significance between the different subtypes of DO in terms of demographics, underlying pathophysiology, symptomology, urodynamic parameters and response to treatment.

## 2 Methods

### 2.1 Study Strategy

This study was conducted following the Preferred Reporting Items for Systematic Reviews and Meta‐analyses (PRISMA) reporting guidelines and registered in the the Open Science Framework (https://doi.org/10.17605/OSF.IO/7VFTU)l [10, 11]. An electronic, systematic search of PubMed, EMBASE, Cochrane, Web of Science and MEDLINE in February 2024. Further details of the search strategy are in the Appendix S1. Two reviewers (M.T. and N.F.W.) screened the identified studies. Any disagreements were resolved by discussion.

### 2.2 Inclusion Criteria

Studies evaluating phasic and terminal DO in humans, including both adults and children, original papers, conference abstracts and English language were included. Only studies that explicitly distinguished between phasic and terminal DO were included. Duplicates, non‐English language, nonhuman studies, studies not reporting original data, case reports and guidelines were excluded.

### 2.3 Data Extraction and Synthesis

Literature search records were systematically retrieved and subsequently imported into EndNote 21.2.0.17387 software and duplicates were removed. Data presented as conference abstracts were also included. Data were extracted into a Microsoft Excel 2016 MSO (Version 2410 Build 18129.20158) spreadsheet which included the following information: first author; year of publishing; location of study; type of study; patient characteristics; inclusion criteria; exclusion criteria; intervention; outcome measure; neurological disease; and summary of results.

### 2.4 Study Risk of Bias Assessment

The Newcastle Ottawa scale critical appraisal tool for cohort studies was utilised to assess the study risk of bias for each of the final 16 studies selected for this systematic review. This assessment can be found in the Appendix S2.

## 3 Results

### 3.1 Study Selection

**FIGURE 1:**
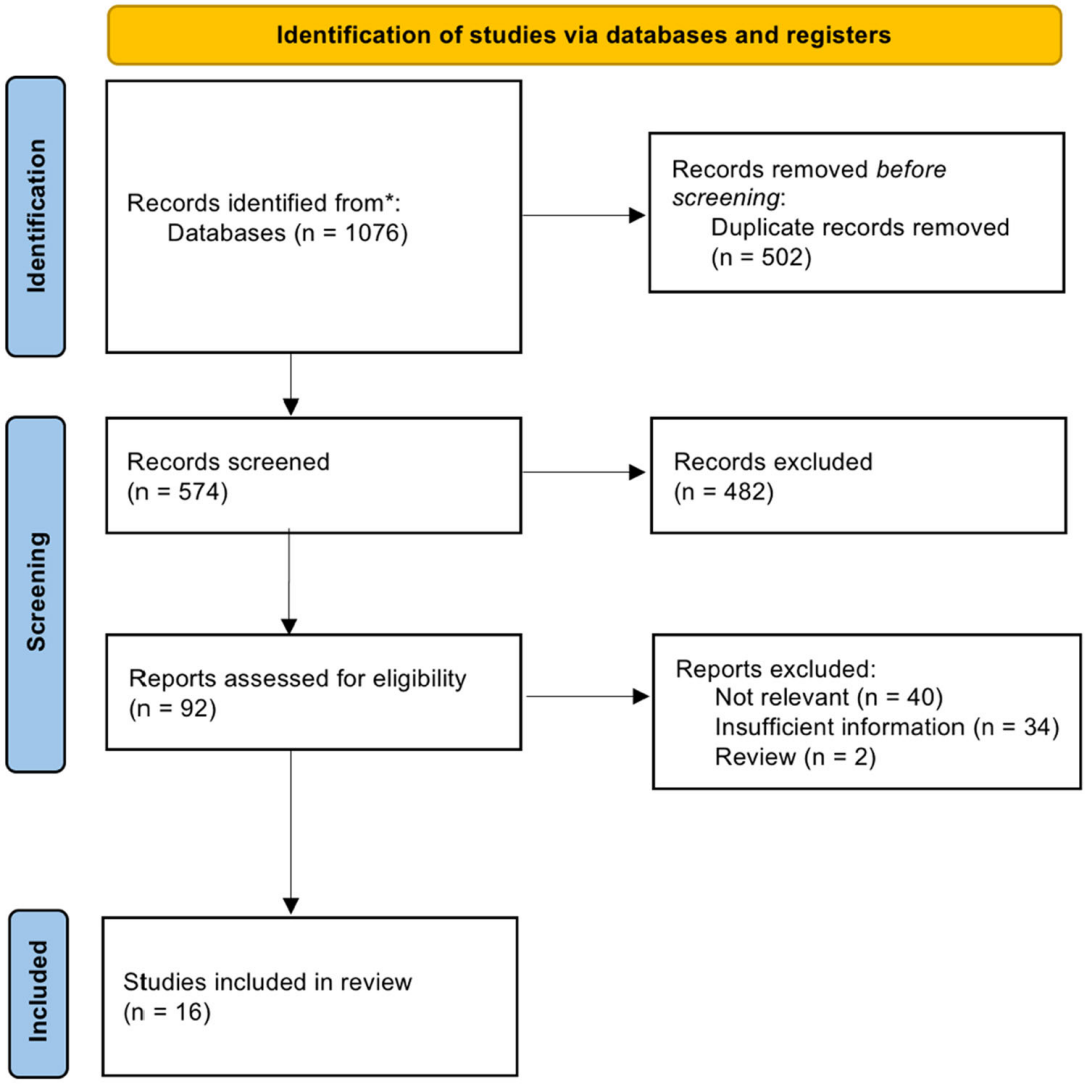
A flowchart showing the results of the study selection process. This was completed following the PRISMA guidelines [11].

The literature search identified 1076 records from electronic databases including PubMed, EMBASE, Cochrane, Web of Science and MEDLINE. The study selection process is illustrated in Figure 1. The search terms used included the

MeSH terms, “detrusor overactivity,” “phasic,” “end fill,” and “terminal.” This comprehensive search yielded a total of 1076 papers: 148 from MEDLINE, 233 from PUBMED, 343 from EMBASE, 41 from Cochrane, and 311 from Web of Science. During the initial screening phase, 502 duplicate records were removed. Subsequently, the remaining 574 records underwent screening in adherence to the predefined exclusion criteria, leading to the exclusion of 334 records. The remaining 92 records underwent a comprehensive full‐text retrieval and screening process. Within this, 40 records were omitted for lack of relevance (studies that did not include end fill, terminal or phasic DO, nonhuman studies, studies analysing tissue samples and cellular level studies) 34 records were omitted due to insufficient information and an additional two records were excluded as review articles. As a result, a total of 16 records were selected for the systematic review.

### 3.2 Study Characteristics

Studies were sorted out into five groups: Associations of PDO and TDO (including sex, age and neurological disease); Symptomology of PDO and TDO; other urodynamic parameters; response of PDO and TDO to oral and intravesical pharmacological treatment; and response of PDO and TDO to surgical treatment. The data tables for each of these study characteristics can be found in the Appendices S3–S6 [12, 13, 14, 15, 16, 17, 18, 19, 20, 21, 22, 23, 24, 25, 26, 27].

### 3.3 Outcomes

#### 3.3.1 Demographics

The search identified seven studies that explored demographic factors associated with the different subtypes of DO.

Across the studies, age was consistently identified as a risk factor for TDO, with TDO patients generally being older than those with PDO. In Cubuk et al. (2021)'s study, patients with TDO were significantly older than patients with PDO (mean age 62.4 vs. 49.9, *p* = 0.039) [13]. Valentini et al. (2011) also reported a similar trend, with TDO patients having a mean age of 63 years compared to PDO patients with a mean age of 52 (*p* = 0.0003) [15].

There was no consistent difference of the prevalence of TDO and PDO between males and females across the seven studies. Cubuk et al. (2021) did not find any statistical gender difference whereas, Vella et al. (2014) reported a higher proportion of males with TDO (26 males vs. six females) [13, 23].

Regarding neurological disease, the studies varied. While Cubuk et al. (2021) found no significant differences in the prevalence of TDO and PDO in patients with any neurological disease, a higher proportion of TDO was observed in those with spinal cord injury (SCI) compared to other neurogenic diseases, although PDO remained more common than TDO within the SCI group of patients (26 vs. 5) [13]. Conversely, Valentini et al. (2011) reported a higher association between TDO and neurological disease, however statistical significance was not achieved [15].

#### 3.3.2 Symptomology

Two studies assessed symptomology in PDO and TDO patients. Urgency and incontinence symptoms were more commonly reported in TDO patients. Zhao et al. (2014) found that patients with preoperative TDO exhibited significantly higher OAB symptom scores than those with PDO, with the TDO subgroup notably more prone to experiencing urgency urinary incontinence [14]. Similarly, Song et al. (2014) noted that children with TDO were more likely to complain of urgency urinary incontinence (UUI) than those with PDO, while no patients with PDO reported urgency or UUI [27]. These findings suggest that TDO may be associated with more severe symptomology. In contrast, patients with PDO across studies were less likely to report urgency or incontinence, highlighting distinct symptom patterns between the two subtypes of DO.

#### 3.3.3 Urodynamic Parameters

Two studies explored the relationship between different urodynamic parameters and the presence of PDO and TDO. Cubuk et al. (2021) found that patients with TDO had a smaller maximum bladder capacity (385.1 mL vs. 463.0 mL, *p* = 0.049) and an earlier first desire to void and strong desire to void (128.8 mL vs. 201.4 mL, *p* = 0.039 and 233.0 mL vs. 328.5 mL, *p* = 0.024, respectively) [13]. Similar findings were observed in the study by Valentini et al. (2011). In this study, females with TDO had smaller functional bladder capacities than women with PDO (246 mL vs. 296 mL, *p* = 0.0045) [15]. These findings suggest that TDO is associated with both a reduced bladder capacity and a lower volume threshold for patient desire to void.

In addition, peak DO amplitude was significantly higher in TDO patients, with Cubuk et al. (2021) reporting a peak DO amplitude of 52.6 cmH_2_0 in TDO patients compared to 16.3 cmH_2_0 those with PDO, (*p* = 0.001) [13].

#### 3.3.4 Responses to Oral Pharmacological Treatment

Two studies evaluated the responses to oral pharmacological treatments in PDO and TDO patients, including antimuscarinics and beta‐3 agonists. Patients with PDO exhibited a higher success rate with oral pharmacological treatment compared to those with TDO. Bharat et al. (2023) found that there was a higher success rate of 60% in patients with PDO than those with TDO when treated with solifenacin, mirabegron, or a combination of both drugs. A successful treatment was defined using a “symptom based scale” [24]. Wang et al. (2022) reported similar findings, with PDO patients demonstrating superior success rates of 50.7% compared to 42% in TDO patients when treated with solifenacin, mirabegron, or combination of both. Mirabegron was found more effective in TDO patients, with a success rate of 50.9% compared to 31.7% for solifenacin and 29.7% for combination therapy (*p* = 0.002). Success was defined as “an improvement in urgency severity and a global response assessment over 1.” [12] These results that beta‐3 agonists, may offer better outcomes for TDO patients, though the overall success rate with pharmacological agents remains higher for patients with PDO than those with TDO.

#### 3.3.5 Responses to Intravesical Treatment

OnabotulinumtoxinA injections were associated with higher success rates in patients with PDO than those with TDO. Ke et al. (2012) reported that 84% of PDO patients experienced improvement in their symptoms following 100U of OnabotulinumtoxinA, compared to 73% of TDO patients in the study (*p* = 0.05). Successful treatment was defined as an improvement in the patient perception of bladder condition (PPBC) questionnaire score over 2. However, there was no significant difference observed in the bladder capacity or Detrusor pressures between the two subtypes of DO following OnabotulinumtoxinA injections [20].

#### 3.3.6 Responses to Surgical Treatment

Five studies evaluated the outcomes of bladder outflow surgery, specifically transurethral resection of the prostate (TURP) and holmium laser enucleation of the prostate (HoLEP) in patients with DO. Across studies, TDO was associated with poorer surgical outcomes compared to PDO.

In TURP studies, Gharib et al. (2022) observed that TDO was more prevalent in patients with persistent storage symptoms following surgery (26.3% vs. 8.9%, *p* = 0.01). Zhao et al. (2014) also reported that OAB symptom scores were less improved in TDO patients (66.7%) compared to PDO patients (79.1%) and patients with TDO were more likely to experience persistent incontinence after surgery (42.9% vs. 7.7%, *p* = 0.037) [18].

Similarly, outcomes following the HoLEP procedure were found to be worse for TDO patients. Kim et al. (2023) found that patients with TDO were more likely require OAB medications within the first 3 months after HoLEP (*p* = 0.003), indicating that TDO may be associated with a less favourable surgical outcome. Criteria to prescribe medication therapy following HoLEP was if “the patient reported further response of urgency occurring more than once a week, experienced additional OAB symptoms, or was discomforted by the symptoms.” Choo et al. (2014) identified that high amplitude DO and TDO were independent risk factors for postoperative UUI [25].

In contrast, PDO patients generally had better outcomes following both TURP and HoLEP procedures across studies, with Alloussi et al. (2013) noting a significantly higher success rate in patients with PDO (69.2%) compared to those with TDO (9.5%) [26].

## 4 Discussion

This systematic review evaluated data from 16 studies, with a total of 3380 patients, and has provided evidence that patients with TDO typically experience more severe symptoms and exhibit less favourable responses to both medical and surgical treatments compared to those with PDO. Specifically, patients with TDO were found to have higher OAB symptom scores and were more likely to report urgency and incontinence, demonstrating a more severe symptom profile. Furthermore, TDO patients demonstrated poorer responses to oral pharmacological therapies such as antimuscarinics, beta‐3 agonists and intravesical treatments such as OnaBTA with PDO patients exhibiting higher success rates. Additionally, following bladder outflow obstruction surgery, patients with preoperative TDO were more likely to have persistent symptoms and require further pharmacological treatment following surgery than those with PDO. These findings suggest that TDO and PDO may have different physiological origins, which may account for the difference observed in symptom severity and treatment outcomes.

The observed clinical differences between TDO and PDO likely arise from underlying differences in pathophysiology. Several studies included in this review highlight that patients with TDO tend to have smaller functional bladder capacities and higher detrusor pressures compared to those with PDO. For example, Cubuk et al. (2021), reported that peak detrusor overactivity amplitude was significantly higher in TDO patients (52.6 cmH_2_O) compared to those with PDO (16.3 cmH_2_O, *p* = 0.001), suggesting that TDO may be associated with a more forceful and abrupt loss of bladder control. Similarly, a lower threshold for the first desire to void in TDO patients supports the notion of increased bladder sensitivity and dysfunction in this group. These objective findings provide a physiological basis for the symptom severity and treatment resistance observed in TDO patients.

The pathophysiology of DO is complex and involves both neural and myogenic mechanisms that are dysregulated with a lack of coordination between the bladder and sphincter leading to premature detrusor contractions in patients [28, 29].

Normal micturition is controlled by the pontine micturition centre (PMC) and consists of two distinct phases: storage and voiding [29]. During the storage phase, the detrusor muscle is relaxed while the urethral sphincter contracts and remains closed to prevent leakage of urine. This action is mediated by the sympathetic nervous system. β‐adrenergic receptors inhibit the contraction of the detrusor muscle, and signals of the hypogastric nerves to the α‐adrenergic receptors maintain contraction of the urethral sphincter [28, 29].

Distension of the bladder wall activates the stretch receptors that transmit signals to the spinal cord. These signals engage the PMC and the periaqueductal grey (PAG). These brain regions play a vital role within the conscious regulation of micturition, particularly providing inhibitory control to prevent premature detrusor contractions during the storage phase [30]. Once the bladder fullness reaches a particular threshold, parasympathetic signals from the spinal cord are transmitted via the pelvic nerve and release acetylcholine, which binds to the M3 muscarinic receptors on the detrusor muscle, leading to contraction of the detrusor muscle. Simultaneously, there is sympathetic inhibition of the urethral sphincter allowing coordinated urine flow [28, 29].

TDO is characterised by “a single voluntary detrusor contraction that cannot be suppressed and that occurs when maximum bladder capacity is reached, resulting in urinary incontinence” [1]. This suggests a pathophysiology involving a failure of the higher cortical centres to inhibit the micturition reflex, particularly the role of the prefrontal cortex and PAG in controlling the “off‐switch” mechanism for the bladder contractions. In cases where these neural connections are disrupted, the detrusor muscle contracts prematurely when bladder volume reaches a critical threshold, leading to early and uncontrolled micturition. In TDO, the loss of supraspinal inhibitory control triggers activation of the PMC in an abrupt and sustained manner, in contrast to the gradual buildup of contractions seen in PDO. Consequently, TDO may result in rapid and complete bladder emptying.

However, it is unlikely that all TDO cases are neurogenic in origin. While conditions such as stroke or neuro‐regenerative diseases may lead to impaired suprapontine inhibition, some cases of TDO in patients may be driven by peripheral bladder afferent dysfunction rather than central impairment.

The International Consultation on Incontinence – Research Society (ICI‐RS) working group previously concluded that there was insufficient evidence to suggest that different patterns of DO have significant clinical relevance [31]. Their review highlighted the limitations of existing studies and the need for robust research. However, since its publication, six additional studies have been conducted, which were included in our review. These newer studies provide further information to suggest that TDO and PDO may represent distinct clinical profiles, with TDO patients experiencing more severe symptoms and poorer responses to both pharmacological and surgical treatments.

This systematic review highlights the importance of differentiating between the pathophysiology of TDO and PDO when evaluating patients' symptoms and when evaluating and deciding their treatment. The distinct pathophysiology underlying each subtype of DO requires tailored therapeutic approaches, as different pharmacological agents can have significantly varied effects on neuronal pathways and receptors depending on the specific form of DO. Recognising these differences is importantly for optimising treatment efficacy and improving patient outcomes, as currently there are no distinct pathways or specific recommendations provided by regulatory bodies and organisations that are tailored for PDO and TDO.

### 4.1 Limitations

Despite the findings presented in this review, several limitations must be acknowledged. The heterogenous nature of the included students, particularly of the patient populations must be taken into account when interpreting the findings, as it may confound the results and limit generalisability. Additionally, most studies relied on retrospective data, which introduces potential selection bias, recall bias and missing data all of which weakens the strength of the conclusions drawn. A further limitation is the variability in how treatment success was defined across studies. Success was primarily based on symptom improvement rather than objective urodynamic measures, and different studies used varying symptom score assessments, making direct comparisons challenging. The absence of randomised controlled trials specifically addressing the clinical significance of DO subtypes prevents definitive casual inferences regarding the symptom severity, treatment outcomes, and the underlying mechanisms of TDO and PDO.

## 5 Conclusion

Patients with terminal DO are more likely to be older, suffer with worse symptoms, have smaller bladder capacities and respond less favourably to treatment than those with phasic DO. Future studies incorporating DO should consider stratifying DO patients into TDO and PDO and assessing them as separate clinical entities.

## Clinical Trial Registration

No clinical trial registration was required for this study as it is a systematic review.

## Ethics Statement

Audit proposal was approved by relevant hospital department.

## Consent

Patient consent was not required for this study. Materials from other sources were not included in the manuscript of this study.

## Conflicts of Interest

The authors declare no conflicts of interest.

## Supporting information

Supplementary 1.

Supplementary 2.

## Data Availability

The data that support the findings of this study are available in the Supporting Information of this article.
