# Supplementary material for: The Clinical Significance of the Subtypes of Detrusor Overactivity: A Systematic Review
Source: Neurourol Urodyn. 2025 Jul 3;44(7):1484–90. doi: 10.1002/nau.70110 (PMC12319479; doi:10.1002/nau.70110)
Supplement: Supplementary file 1 — Supplementary 1. [file NAU-44-1484-s002.docx]

Appendix (1): Search Strategy

|  | **MEDLINE ALL** | **PUBMED** | **EMBASE** | **Cochrane** | **Web of Science** |
| --- | --- | --- | --- | --- | --- |
| **1** | Detrusor overactivity OR Overactive Bladder OR Overactive Urinary Bladder OR Bladder, Overactive OR Overactive Detrusor OR Detrusor, Overactive OR Overactive Detrusor Function OR Detrusor Function, Overactive OR Detrusor Hyperreflexia | Detrusor overactivity OR Overactive Bladder OR Overactive Urinary Bladder OR Bladder, Overactive OR Overactive Detrusor OR Detrusor, Overactive OR Overactive Detrusor Function OR Detrusor Function, Overactive OR Detrusor Hyperreflexia | Detrusor overactivity OR Overactive Bladder OR Overactive Urinary Bladder OR Bladder, Overactive OR Overactive Detrusor OR Detrusor, Overactive OR Overactive Detrusor Function OR Detrusor Function, Overactive OR Detrusor Hyperreflexia | Detrusor overactivity OR Overactive Bladder OR Overactive Urinary Bladder OR Bladder, Overactive OR Overactive Detrusor OR Detrusor, Overactive OR Overactive Detrusor Function OR Detrusor Function, Overactive OR Detrusor Hyperreflexia | Detrusor overactivity OR Overactive Bladder OR Overactive Urinary Bladder OR Bladder, Overactive OR Overactive Detrusor OR Detrusor, Overactive OR Overactive Detrusor Function OR Detrusor Function, Overactive OR Detrusor Hyperreflexia |
| **#** | 11,688 | 12,129 | 23,804 | 3,743 | 16,498 |
| **2** | Phasic | Phasic | Phasic | Phasic | Phasic |
| **#** | 16,986 | 17,393 | 20,536 | 704 | 15,444 |
| **3** | End fill | End fill | End fill | End fill | End fill |
| **#** | 31 | 2,265 | 58 | 1,898 | 30,509 |
| **4** | Terminal | Terminal | Terminal | Terminal | Terminal |
| **#** | 486,398 | 677,632 | 658,440 | 12,297 | 656,611 |
|  | | | | | |
| **1 AND (2 OR 3 OR 4)** | **148** | **233** | **343** | **41** | **311** |

Total number of papers identified: 1076

Appendix (2): Summary of selected papers

1. Alloussi S, Peters G, Al-Bulushi Y. Can we forecast the postoperative results in patients with bladder outlet obstruction (boo)? European Urology. 2013 Mar;12(1, Supplement):e514
2. Balsamo R, Uricchio F, Costantini E, De Sio M, Illiano E, Arcaniolo D, et al. Anterior colporrhaphy and sacrospinous hysteropexy in women with pelvic organ prolapse: Urodynamic findings and functional outcomes. European Journal of Obstetrics & Gynecology and Reproductive Biology [Internet]. 2022 [cited 2024 Mar 2];271:255–9. Available from: <https://linkinghub.elsevier.com/retrieve/pii/S0301211522000756>
3. Bharat P, Saravanan K, Sundar V, Sarvanan P, Santhaseelan W. Urodynamic findings affecting the success of medical management in patients of overactive bladder: A prospective observational study. Indian Journal of Urology. 2023 Jan;39(5, Supplement):S22.
4. Choo MS, Kim M, Lee HE, Oh SJ. LARGE DETRUSOR OVERACTIVITY PREDISPOSES URGENCY URINARY INCONTINENCE AFTER HOLEP IN PATIENTS WITH BENIGN PROSTATIC HYPERPLASIA. Journal of Urology [Internet]. 2014 [cited 2024 Mar 1];191(4S). Available from: <http://www.jurology.com/doi/10.1016/j.juro.2014.02.543>
5. Çubuk A, Şahan A, Özkaptan O, Simsek B, Sulukaya M, Tarhan F. The types of urodynamic detrusor overactivity and its relationship with neurological diseases. 10-years follow-up of 1000 invasive urodynamic studies. Eastern J Med [Internet]. 2021 [cited 2024 Mar 2];26(1):139–44. Available from: <https://jag.journalagent.com/z4/download_fulltext.asp?pdir=ejm&plng=eng&un=EJM-92678>
6. De Ridder D, Chandiramani V, Dasgupta P, Van Popple H, Baert L, Fowler CJ. INTRAVESICAL CAPSAICIN AS A TREATMENT FOR REFRACTORY DETRUSOR HYPERREFLEXIA: A DUAL CENTER STUDY WITH LONG-TERM FOLLOWUP. Journal of Urology [Internet]. 1997 [cited 2024 Mar 2];158(6):2087–92. Available from: <http://www.jurology.com/doi/10.1016/S0022-5347%2801%2968162-X>
7. Gharib T, Eldakhakhny A, Alazaby H, Khalil M, Elgamal K, Alhefnawy M. Evaluation of Storage Symptoms Improvement and Factors Affecting, After Relief of Obstruction in Patients With Benign Prostatic Enlargement. Urology [Internet]. 2022 [cited 2024 Mar 2];169:180–4. Available from: <https://linkinghub.elsevier.com/retrieve/pii/S0090429522005969>
8. Grigoleit U, Mürtz G, Laschke S, Schuldt M, Goepel M, Kramer G, et al. Efficacy, Tolerability and Safety of Propiverine Hydrochloride in Children and Adolescents with Congenital or Traumatic Neurogenic Detrusor Overactivity—A Retrospective Study. European Urology [Internet]. 2006 [cited 2024 Mar 2];49(6):1114–21. Available from: <https://linkinghub.elsevier.com/retrieve/pii/S0302283806002041>
9. Ke QS, Chen YC, Kuo HC. Do baseline urodynamic parameters affect the treatment outcome after intravesical 100 U onabotulinumtoxinA injection in patients with idiopathic detrusor overactivity? Tzu Chi Medical Journal [Internet]. 2012 [cited 2024 Mar 2];24(3):121–6. Available from: <http://linkinghub.elsevier.com/retrieve/pii/S1016319012000043>
10. Kim SJ, Park SG, Pak S, Kwon O, Lee YG, Cho ST. Predictive factors for postoperative medication therapy for overactive bladder symptoms after holmium laser enucleation of prostate. Int J of Urology [Internet]. 2023 [cited 2024 Mar 2];30(11):1036–43. Available from: <https://onlinelibrary.wiley.com/doi/10.1111/iju.15260>
11. Song S, Lee D, Sohn M, Hong B. A pattern of detrusor overactivity in urodynamic analysis is associated with lower urinary tract symptoms but not with renal function deterioration in neurogenic bladder dysfunction. Urology [Internet]. 2014 Oct 1 [cited 2024 Mar 1];84(4, Supplement):S1–146. Available from: <https://www.sciencedirect.com/science/article/pii/S0090429514010176>
12. Valentini FA, Marti BG, Robain G, Nelson PP. Phasic or terminal detrusor overactivity in women: age, urodynamic findings and sphincter behavior relationships. Int braz j urol [Internet]. 2011 [cited 2024 Mar 2];37(6):773–80. Available from: <http://www.scielo.br/scielo.php?script=sci_arttext&pid=S1677-55382011000600014&lng=en&tlng=en>
13. Valentini F, Marti B, Robain G. DETRUSOR OVERACTIVITY: DO THE DIFFERENT PATTERNS HAVE CHARACTERISTICS RELATED TO GENDER OR CLINICAL CONDITION? Journal of Urology [Internet]. 2013 [cited 2024 Mar 2];189(4S). Available from: <http://www.jurology.com/doi/10.1016/j.juro.2013.02.2196>
14. Vella M, Gattuso S, Romeo S, D’Amato F. Urodynamics and overactive bladder (OAB). What’s the incidence of detrusorial overactivity (DO) and what’s the role of isometric detrusorial pressure? A prospective study. Neurourology and Urodynamics [Internet]. 2014 [cited 2024 Mar 2];33(S2):S54–5. Available from: <https://onlinelibrary.wiley.com/doi/10.1002/nau.22620>
15. Wang HJ, Kuo HC. Effects of different urodynamic characteristics on therapeutic outcomes of overactive bladder medication in a real-life clinical practice. Tzu Chi Medical Journal [Internet]. 2022 Dec [cited 2024 Mar 2];34(4):441. Available from: <https://journals.lww.com/tcmj/fulltext/2022/34040/effects_of_different_urodynamic_characteristics_on.14.aspx>
16. Zhao Y, Liu W, Guralnick M, Niu W, Wang Y, Sun G, et al. Predictors of short‐term overactive bladder symptom improvement after transurethral resection of prostate in men with benign prostatic obstruction. Int J of Urology [Internet]. 2014 [cited 2024 Mar 2];21(10):1035–40. Available from: <https://onlinelibrary.wiley.com/doi/10.1111/iju.12482>
