# Supplementary material for: The Clinical Significance of the Subtypes of Detrusor Overactivity: A Systematic Review
Source: Neurourol Urodyn. 2025 Jul 3;44(7):1484–90. doi: 10.1002/nau.70110 (PMC12319479; doi:10.1002/nau.70110)
Supplement: Supplementary file 2 — Supplementary 2. [file NAU-44-1484-s001.docx]

Appendix (2): Newcastle Ottawa Risk of Bias Assessment

	Song et al. 2014	Alloussi et al. 2013	Valentini et al. 2013	Choo et al. 2014	Bharat et al. 2023	Vella et al. 2014	Balsamo et al. 2022	Ke et al. 2012	Wang et al. 2022	Grigoleit et al. 2006	Gharib et al. 2022	De Ridder et al. 1997	Valentini et al. 2011	Kim et al. 2024	Zhao et al. 2014	Cubuk et al. 2021
**Selection**																
Representativeness of the exposed cohort																
(a) Truly representative	*	*	*	*	*	*	*	*	*	*	*	*	*	*	*	*
(b) Somewhat representative																
Selection of non exposed cohort																
(a) Drawn from same community as exposed cohort	*	*	*	*	*	*	*	*	*	*	*	*	*	*	*	*
Ascertainment of exposure																
(a) Secure record (urodynamics)	*	*	*	*	*	*	*	*	*	*	*	*	*	*	*	*
Demonstration that outcome of interest was not present at start of the study																
(a) Yes	*	*	*	*	*	*	*	*	*	*	*	*	*	*	*	*
**Comparability**																
(a) Patients with Phasic DO compared with End Fill DO	*	*	*	*	*	*	*	*	*	*	*	*	*	*	*	*
**Outcome**																
Assessment of outcome																
(a) Independent blind linkage																
(b) Record linkage	*	*	*	*	*	*	*	*	*	*	*	*	*	*	*	*
Was follow up long enough for outcomes to occur?																

(a) Yes	*	*	*	*	*	*	*	*	*	*	*	*	*	*	*	*
(b) No																
Adequacy of follow up																
(a) Complete follow up – all subjects accounted for		*		*	*		*	*	*	*	*	*		*	*	
(b) Subjects lost to follow up – unlikely to introduce bias																
(c) Follow up rate less than 80% and no description of those lost																
(d) No statement	*		*			*							*			*
**Total number of stars:**	7	8	7	8	8	7	8	8	8	8	8	8	7	8	8	
**Quality:**	*Good*	*Good*	*Good*	*Good*	*Good*	*Good*	*Good*	*Good*	*Good*	*Good*	*Good*	*Good*	*Good*	*Good*	*Good*	*Good*

Appendix (3): Data Table for Demographics

*End fill detrusor overactivity is often referred to as terminal detrusor overactivity (TDO).

**Author, Year, Country & Publication Type**	**Patient Characteristics**	**Inclusion criteria**	**Exclusion criteria**	**Intervention**	**Outcome Measure**	**Neurological Disease?**	**Results**
Song et al., 2014, Republic of Korea, Abstract	48 patients<br>DO in 83.3% of patients<br><br>Age: 10.83 ± 5.58	Diagnosis of neurogenic bladder	N/A	Urodynamic Test	Urodynamics	Yes	Phasic DO in 77.5%.<br>Terminal DO in 22.5% .<br><br>Terminal DO complain of urgency or urge incontinence more frequently than Phasic DO.
Vella et al., 2014, Italy, Abstract	32 consecutive patients.<br><br>26 Male (median: 67.3)<br>6 Female (median: 51.5)	Patients with overactive bladder	Urinary infections, bladder tumour, XR therapy, urolithiasis, neurological diseases, pelvic prolapses etc.	Urodynamics	Voiding diary, Urodynamics	None	Phasic DO in 4 (12.5%) patients.<br>Terminal DO in 8 (25%) patients.<br>Both in 9 (28.1%) patients.
Cubuk, 2021, Turkey, Full Paper	1018 patients<br>619 men<br>409 women<br><br>PDO: 837 (252M/585F)<br>Age: 49.9±15.5<br><br>TDO: 45 (17M/28F)<br>Age: 62.4±7.9	Patients with detrusor overactivity	N/A	Urodynamic Test	Urodynamic	Yes	First desire to void is larger in PDO than TDO ($p = 0.032$)<br>Maximum bladder capacity larger in PDO than TDO ($p = 0.001$)<br>Amplitude of DO is larger in TDO than PDO ($p = 0.001$)<br>Spinal Cord Trauma is associated with higher rates of TDO than PDO ($p = 0.04$)
Valentini et al., 2013, France, Abstract	203 patients with detrusor overactivity	Patients with detrusor overactivity	N/A	Urodynamics	Urodynamics	None	Phasic DO patients significantly younger

							than population (p<0.0001).<br>Frequency of urgency is similar in phasic and terminal DO.<br>Phasic DO occurs in younger patients.
Valentini, 2011, France, Full Paper	164 successive women referred for LUTS with diagnosis DO<br><br>Age Range: 18 – 75+	Patients with phasic or terminal DO with LUTS symptoms	complete spinal cord injury, severe dementia (mini mental state <20) and grade ≥ 2 prolapse)	Medical history, medication, bladder diary and urodynamics	Cystometry, bladder diary, physical examination	Yes	Occurrence: PDO (77) vs TDO (87)<br>Age: PDO patients younger (p = 0.0003)<br>Neuro disease: TDO more frequency with neuro disease.<br>Age trend: PDO constant; TDO increases with age (6.7% to 23.3%, p=0.0013)<br>Bladder capacity: Higher in PDO than TDO

Appendix (4): Data Table for symptomology of phasic and end fill detrusor overactivity

Author, Year, Country & Publication Type	Patient Characteristics	Inclusion criteria	Exclusion criteria	Intervention	Outcome Measure	Neurological Disease?	Results
Choo et al., 2014, Republic of Korea, Abstract	409 male patients total. 180 patients with involuntary detrusor contractions – Phasic DO: 83, Terminal DO: 57, Both: 68	Benign Prostate Hyperplasia requiring HoLEP (holmium laser enucleation of prostate)	N/A	HoLEP – Holmium laser enucleation of the prostate	Urodynamics – presence of UUI	None	Age, Terminal IDC, and urine leakage during filling cystometry were interdependent risk factors of UUI at immediate post operative period (p=0.055).
Zhao et al., 2014, China, Full Paper	128 male patients<br>TDO: 18<br>(14 moderate, 4 severe based on OABSS)<br>PDO: 25	Urgency symptoms, i proven BOO, OABSS	Age <50 years, PV <20 mL or >150 mL, urgency score <2 (OABSS), non-BPH BOO, prostate carcinoma, prior prostatic/urethral surgery, bladder neoplasm/stones, UTI/chronic prostatitis, neurogenic bladder, or recent anticholinergic/sympathomimetic use	TURP	OABSS and Urodynamics	None	Preoperative OABSS: Higher in Terminal DO vs Phasic DO and no DO (p<0.05)<br>Urge incontinence: more frequent TDO (P<0.001)<br>OABSS Pre and Post TURP: Higher in Terminal DO vs Phasic DO (p<0.01) and non-DO (p<0.05)<br>Treatment success (over 50% OABSS improvement): Lower in terminal DO (66.7%) vs non TDO (90.9%) (p<0.05)
Valentini, 2011, France, Full Paper	164 successive women referred for LUTS with diagnosis DO<br><br>Age Range: 18 – 75+	Patients with phasic or terminal DO with LUTS symptoms	complete spinal cord injury, severe dementia (mini mental state <20) and grade ≥ 2 prolapse)	Medical history, medication, bladder diary and urodynamics	Cystometry, bladder diary, physical examination	Yes	Occurrence: PDO (77) vs TDO (87)<br>Age: PDO patients younger (p = 0.0003)<br>Neuro disease: TDO more frequency with neuro disease.<br>Age trend: PDO constant; TDO increases with age (6.7% to 23.3%, p=0.0013)<br>Bladder capacity: Higher in PDO than TD
Cubuk, 2021, Turkey, Full Paper	1018 patients<br>619 men<br>409 women	Patients with detrusor overactivity	N/A	Urodynamic Test	Urodynamic	Yes	First desire to void is larger in PDO than TDO (p = 0.032)<br>Maximum bladder capacity larger in PDO than TDO (p = 0.001)

	PDO: 837 (252M/585F)<br>Age: 49.9±15.5<br><br>TDO: 45 (17M/28F)<br>Age: 62.4±7.9						Amplitude of DO is larger in TDO than PDO ($p = 0.001$)<br>Spinal Cord Trauma is associated with higher rates of TDO than PDO ($p = 0.04$)

Appendix (5): Data Table for responses of phasic and end fill detrusor overactivity to pharmacological treatment

Author, Year, Country & Publication Type	Patient Characteristics	Inclusion criteria	Exclusion criteria	Intervention	Outcome Measure	Neurological Disease	Results
Bharat et al., 2023, India, Abstract	150 patients	Patients with overactive bladder and Urodynamic Detrusor overactivity	N/A	Patients started on solifenacin, mirabegron or combination therapy of both for 1-3 months	Urodynamics	Yes	Phasic DO (60%) had higher success rates than those with Terminal DO
Ke et al., 2012, Taiwan, Full Paper	174 patients<br>89 men, 85 women<br><br>Age: Range 18 – 94 years old, Median of 67 years	Urodynamic DO with or without urinary incontinence refractory to previous antimuscarinics for more than 3 months and no UTI, stress incontinence, BOO or neurogenic bladder at enrolment	N/A	First single intravesical BoNT-A 100 U	Improvement of at least two points on a patient perception bladder condition scale<br>Urodynamics	No	No significant difference was found between the cumulative success rates between any patient subgroup of Phasic and Terminal DO on a long-term basis.<br>At 3 months, Phasic DO has a significantly higher success rate than terminal DO at 3 months.
Wang et al., 2022, Taiwan, Full Paper	453 consecutive patients<br>315 male and 138 women<br><br>TDO: 305, PDO: 148<br><br>Mean Age: 70.1 ± 12.3 years	Symptoms of urgency or frequency with or without UUI, patients who have undergone VUDS conforming Urodynamic DO	N/A	Patients given either mirabegron, solifenacin or combination therapy with both if first treatment is proven ineffective and	Urodynamics, Success or failure of overactive bladder	Yes	Patients initially treated with solifenacin - 144, mirabegron - 255, and a combo of solifenacin and mirabegron - 54. Patients with TDO had lower success rates compared to PDO, mirabegron better success rate with OAB patients.

		selected for analysis on the therapeutic efficacy of initial OAB medication, patients who had been previously treated with OAB medication but discontinued for more than 6 months		then tolterodine and oxybutynin. Patients given either mirabegron, solifenacin or combination therapy with both if first treatment is proven ineffective and then tolterodine and oxybutynin			Success Totals:<br>Phasic 50.7% success, Terminal 42.0% success.
Grigoleit et al., 2006, Germany, Full Paper	74 patients both male and female.<br>32 with phasic DO<br><br>Age Range: 11 months to 19 years	Having neurogenic detrusor overactivity and having been allocated to treatment with Propiverine		Propiverine hydrochloride	Urodynamics	No	Phasic DO was abolished in 63% patients
De Ridder et al., 1997, London and Leuven, Full Paper	Dual Centres: London and Leuven<br>London: 30 patients, 11M/11F<br>Age: 14-70 years (Mean: 45)	N/A	N/A	Intravesical instillations of 1 to 2 mol./l. Of capsaicin in 30% ethanol in saline.	Urodynamics	Yes	London:<br>- 18 patients with phasic detrusor hyperreflexia.<br>- Bladder capacity: 169±80 → 320±129 ml.<br>- Max detrusor pressure: 68±29 → 49±28 cmH_2O.<br>- Outcomes: 11 excellent, 3 satisfactory, 4 no improvement.<br><br>Leuven:<br>- 49 patients with phasic detrusor hyperreflexia.<br>- Bladder capacity: 194±82 → 247±96 ml.<br>- Max detrusor pressure: 58±25 → 28±10 cmH_2O.<br>- Outcomes: 13 excellent, 27 satisfactory, 9 unsatisfactory.

							Overall: Repeated instillations effective for 3–5 years. Complete continence: 44%; satisfactory: 36%; failure: 20%.

Appendix (6): Data Table for responses of phasic and end fill detrusor overactivity to surgical treatment

Author, Year, Country & Publication Type	Patient Characteristics	Inclusion criteria	Exclusion criteria	Intervention	Outcome Measure	Neurological Disease	Results
Balsamo et al., 2022, Italy, Full Paper	62 women, Mean Age: 64.4 ± 8.1	Women affected by anterior/central compartment prolapse of stage II-IV undergoing anterior colporrhaphy and bilateral SSH	Previous pelvic organ cancer, connective tissue disorders, neurologic diagnoses, previous POP surgery, previous anti incontinence procedures, previous radial pelvic surgery	Anterior Colporrhaphy and bilateral SSH	UDI-6 score and IIQ-7 scores pre and post op.	No	At baseline, 24 (38.7%) women had typical phasic DO. Six months after surgery, DO detected in 11 women (17.7%) ($p<0.0001$), with no de novo cases.<br>At last follow up visit, UDI-6 and IIQ-7 total median scores improved significantly ($p<0.001$).
Alloussi et al., 2013, Germany, Abstract	103 Male patients diagnosed with BOO<br><br>Age: 69.7±8.2	Overactive Bladder due to Bladder Outlet Obstruction	N/A	TURP	Urodynamics	No	Combined UDS 24 hours pre op and then follow up 3 months post op.<br>Phasic DO success post TURP = 62%,<br>Terminal DO success post TURP = 9.5%
Gharib et al., 2022, Egypt, Full Paper	75 male patients with BPE<br><br>Age:<br>Mean - 67.88 ± 7.82<br>Range - 50-88	Patients with BPE less than 80gm and storage symptoms, indication for TURP following BPE	Neurological disease, prostate cancer, previous prostate surgery, urethral stricture, stone bladder, bladder cancer, acute and chronic prostatitis	TURP	IPSS/Blaivais Score 2007/Digital Prostate exam/PSA	No	- Patients with persistent symptoms were older ($p=0.022$).<br>- Terminal DO more common in persistence group (26.3% vs 8.9%), with higher Qmax.<br>- MCC significantly higher in resolution group ($p=0.001$).<br>- Terminal DO is a significant predictor for persistent storage symptoms post-TURP.
Kim et al., 2023, South Korea, Full Paper	257 male patients<br><br>Mean Age: 71.45	Received BPH pharmacotherapy for at least 1 month	History of BPH surgery, diagnosis of prostate cancer/urethral stenosis, recent urinary tract infection, or pre operative treatment such as sacral neuro modulation or Botox	HoLEP and post op medication	IPSS and Urodynamics	No	Prevalence of TDO significantly higher in medication group than in medication free group n=82 (39.4%) vs (n=29 (63%) $p=0.003$<br>Patients placed in medication group had higher symptom scores.

Zhao et al., 2014, China, Full Paper	128 male patients<br>TDO: 18<br>(14 moderate, 4 severe based on OABSS)<br>PDO: 25	Urgency symptoms, urodynamically proven BOO, OABSS	Age <50 years, PV <20 mL or >150 mL, urgency score <2 (OABSS), non-BPH BOO, prostate carcinoma, prior prostatic/urethral surgery, bladder neoplasm/stones, UTI/chronic prostatitis, neurogenic bladder, or recent anticholinergic/sympathomimetic us.	Transurethral resection of the prostate (TURP)	OABSS and Urodynamics	No	Preoperative OABSS: Higher in Terminal DO vs Phasic DO and no DO ($p<0.05$)<br>Urge incontinence: more frequent TDO ($P<0.001$)<br>OABSS Pre and Post TURP: Higher in Terminal DO vs Phasic DO ($p<0.01$) and non-DO ($p<0.05$)<br>Treatment success (over 50% OABSS improvement): Lower in terminal DO (66.7%) vs non TDO (90.9%) ($p<0.05$)
